# Immunization, urbanization and slums – a systematic review of factors and interventions

**DOI:** 10.1186/s12889-017-4473-7

**Published:** 2017-06-08

**Authors:** Tim Crocker-Buque, Godwin Mindra, Richard Duncan, Sandra Mounier-Jack

**Affiliations:** 1 Health Protection Research Unit in Immunisation, Faculty of Public Health and Policy, London School of Hygiene and Tropical Medicine, 15-17 Tavistock Place, London, WC1H 9SH UK; 2 Programme Division, Health Section, UNICEF Headquarters, 3 United Nations Plaza, New York, 10017 USA

**Keywords:** Vaccine, Immunization, Urban, Slum, Low-income

## Abstract

**Background:**

In 2014, over half (54%) of the world’s population lived in urban areas and this proportion will increase to 66% by 2050. This urbanizing trend has been accompanied by an increasing number of people living in urban poor communities and slums. Lower immunization coverage is found in poorer urban dwellers in many contexts. This study aims to identify factors associated with immunization coverage in poor urban areas and slums, and to identify interventions to improve coverage.

**Methods:**

We conducted a systematic review, searching Medline, Embase, Global Health, CINAHL, Web of Science and The Cochrane Database with broad search terms for studies published between 2000 and 2016.

**Results:**

Of 4872 unique articles, 327 abstracts were screened, leading to 63 included studies: 44 considering factors and 20 evaluating interventions (one in both categories) in 16 low or middle-income countries. A wide range of socio-economic characteristics were associated with coverage in different contexts. Recent rural-urban migration had a universally negative effect. Parents commonly reported lack of awareness of immunization importance and difficulty accessing services as reasons for under-immunization of their children. Physical distance to clinics and aspects of service quality also impacted uptake. We found evidence of effectiveness for interventions involving multiple components, especially if they have been designed with community involvement. Outreach programmes were effective where physical distance was identified as a barrier. Some evidence was found for the effective use of SMS (text) messaging services, community-based education programmes and financial incentives, which warrant further evaluation. No interventions were identified that provided services to migrants from rural areas.

**Conclusion:**

Different factors affect immunization coverage in different urban poor and slum contexts. Immunization services should be designed in collaboration with slum-dwelling communities, considering the local context. Interventions should be designed and tested to increase immunization in migrants from rural areas.

**Electronic supplementary material:**

The online version of this article (doi:10.1186/s12889-017-4473-7) contains supplementary material, which is available to authorized users.

## Background

In 2014, over half (54%) of the world’s population lived in urban areas [1]. By 2050 this proportion is expected to increase to 66%, adding approximately 2.5 billion new urban dwellers, of whom 90% will be in Asia and Africa [2]. This trend towards urbanization is closely linked with an increasing population living in urban poor communities and slum environments [2]. Although the proportion of urban residents living in slums has been decreasing, the absolute number has increased substantially, from 689 million in 1990 to 880 million in 2014, and is expected to double by 2030 [3]. In order to meet the outcomes in the New Urban Agenda and Sustainable Development Goals to improve health for slum-dwellers a better understanding is needed of each individual slum environment and the factors contributing to poor health outcomes [4, 5].

### Immunization and urbanization

**Fig. 1:**
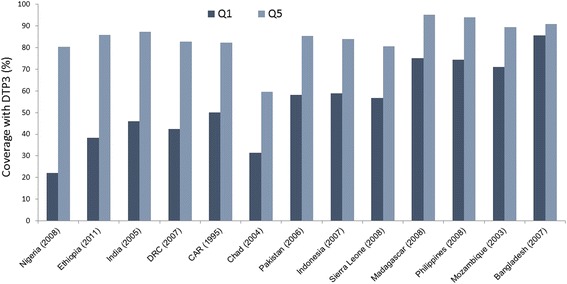
DTP3 coverage in selected countries ranked in order of difference between wealth quintiles DTP3 = 3rd dose of diphtheria, tetanus and pertussis vaccine. Q1 = poorest and Q5 = wealthiest urban wealth quintiles. Data from WHO Observatory [105]

Significant disparities exist in immunization coverage in urban areas with lower coverage observed in the urban poor in many countries (Fig. 1).

Although some countries, such as Bangladesh, have achieved immunization equity, these disparities are growing in many others, including Nigeria, Ethiopia, Cameroon and Pakistan [6]. However, Demographic Health Surveys do not sample urban slums specifically Tables 1 and 2. display the results of recent studies that have reported data on immunization coverage in slums in India and in sub-Saharan African countries, showing a wide range of coverage, from 3.0% and 8.5% in rural migrant children in slums in Chandigarh and Nigeria [7, 8], to 88.7% and 93.3% in Mumbai and Ouagadougou [9, 10].

**Table 1:** Showing studies conducted in slum populations in sub-Saharan Africa reporting immunization status of children since 2000

Ref	First Author	Year	Population	Country	Sample size	Immunization Status (%)			Notes
						Complete	Partial	Unimmunized	
[10]	<<Soura	2015	Children 12–59 months	Burkina Faso	3103	93.3	6.7		
[65]	Maina	2013	Children 12–23 months	Kenya	380	76.6	23.4		
[64]	Egondi	2015	Children 12–23 months	Kenya	382	70.0	30.0		
[73]	Bobossi-Serengibé	2014	Children <11 months	CAR	400	67.0	33.0		
[66]	Mutua	2011	Children 12–23 months	Kenya	1848	58.0	42.0		
[10]	<<Soura	2015	Children 12–59 months	Kenya	1369	55.0	45.0		
[72]	Mohamud	2014	Children 12–23 months	Ethiopia	582	47.6	32.7	19.7	Low-income urban (not slum specifically)
[8]	Fatiregun	2013	Children 12–23 months	Nigeria	588	38.8	45.6	15.7	Low-income urban (not slum specifically)
[71]	^Antai	2012	Children >12 months	Nigeria	604	24.3	75.7		Rural
	^Antai	2012	Children >12 months	Nigeria	593	15.2	84.8		Urban
	^Antai	2012	Children >12 months	Nigeria	1303	8.5	91.5		Rural-Urban migrant

<< and ^ denote results from the same study disaggregated by urban, rural or migration status

**Table 2:** Showing studies conducted in slum populations in India reporting immunization status of children since 2000

Ref	First Author	Year	Population	Location	Sample size	Immunization Status (%)			Notes
						Complete	Partial	Unimmunized	
[9]	Kulkarni	2013	Children 12–23 months	Mumbai	352	88.7	11.9		Complete or incomplete
[101]	Damor	2013	Children 1–5 years	Jamnagar	450	75.0	13.3	11.6	
[39]	Kadarkar	2016	Children 12–23 months	Mumbai	336	75.0	22.3	2.7	
[45]	Trivedi	2014	Children 12–23 months	Rewa	210	72.4	21.9	5.7	
[56]	Kar	2001	Children 12–23 months	South Delhi	166	69.3	15.7	15.1	
[49]	Wadgave	2012	Children <5 years	Solapur	420	64.3	25.6	9.8	
[53]	>Kusuma	2010	Rural-urban migrant children up to 2 years	Delhi	746	60.2	34.9	4.9	Settled migrants
[40]	Awasthi	2015	Children 12–23 months	Varanasi	384	57.0	43.0		Complete or incomplete
[41]	Khan	2015	Children 12–23 months	Jagdalpur	225	55.1	30.7	14.2	
[57]	Desai	2003	Children 9–59 months	Surat	3035	49.3	51.7		Measles only
[50]	Sachdeva	2012	Children 12–23 months	New Delhi	210	47.8	17.2	35.2	Hep B only
[42]	Kulkarni	2014	Children 12–23 months	Hyderabad	510	44.1	32.0	23.9	
[54]	Nath	2007	Children 12–23 months	Lucknow	510	44.1	32.0	23.9	
[44]	Agarwal	2014	Children <5 years	Kanpur	390	41.4	44.8	13.8	
[53]	>Kusuma	2010	Rural-urban migrant children up to 2 years	Delhi	746	39.7	54.8	5.5	Recent migrants
[102]	Gupta	2012	Children <5 years	Bhopal	790	35.2	48.2	n/a	16.4% status unknown
[47]	Angandi	2013	Children 12–23 months	Bijapur	155	34.8	62.6	2.6	
[52]	Jain	2010	Children 12–23 months	Meerut	216	31.0	17.1	51.9	
[103]	Sharma	2009	Children 12–23 months	Surat	300	25.1	51.7	23.1	
[55]	Mathew	2002	Children <5 years	New Delhi	500	25.0	44.4	30.6	
[7]	#Sharma	2015	Children 12–23 months	Chandigarh	310	23.0	73.0	3.0	Non-migrants
[51]	Ghei	2010	Children 10–23 months	Agra	1728	14.0	45.0	41.0	
[7]	#Sharma	2015	Children 12–23 months	Chandigarh	310	3.0	91.0	6.0	Migrants

> and # denote results from the same study disaggregated by migration status

Where immunization coverage is low Vaccine Preventable Diseases (VPDs) contribute to worse health outcomes in poor urban populations, particularly in slums. Increased morbidity and mortality has been observed from measles [11, 12], mumps [13], diphtheria [14], influenza [15], and typhoid [16–19]; in slums in: South Africa [11], India [12, 13, 17, 19], Bangladesh [15, 18] and Kenya [16]. Outbreaks of VPDs are more common in urban slums than in other urban areas and have a larger number of cases secondary to high population density and continuous influx of a new pool of infective agents with migratory populations [11–14, 19]. Crowding is known to increase childhood mortality from VPDs [20, 21].

In addition, vaccine programmes designed for a general population may not be as effective in urban slums, which are characterized by lack of essential infrastructure (such as electricity and water), poor housing quality and where residents may be recent migrants, or have insecure legal or residential status, limiting access to basic health services, if they are available at all [22, 23].

Although low immunization coverage is not inevitable amongst the urban poor, there is no current systematic synthesis describing the associated demographic, geographic, and socio-economic factors, nor considering interventions to increase coverage. Therefore, the aim of this study is to identify the factors associated with immunization coverage in low-income urban areas and slums in low and middle-income countries (LMICs), and identify the evidence for interventions to improve coverage.

## Methods

We conducted a systematic review in line with the Preferred Reporting Items for Systematic Reviews and Meta-Analysis (PRISMA) statement [24].

### Search strategy

We searched Medline, Embase, Global Health, CINAHL, Web of Science and The Cochrane Database of Systematic Reviews, using the strategy shown in Additional file 1.

### Inclusion and exclusion criteria

Studies published between 2000 and July 2016 with the following characteristics were included:

- *Population*: any population living in a low-income urban area or slum in a LMIC [25]. Slums have a broad operational definition, so we have included consideration of urban poor communities that have slum-like characteristics, even if they have not been formally designated as a slum [22].
- *Study design:*
  - *Observational studies:* cross-sectional surveys and cohort studies, designed to identify factors associated with coverage levels.
  - *Intervention studies:* randomised controlled trials (RCTs), quasi-experimental (including time-series and before-and-after studies) and ecological designs that evaluated any intervention designed to increase vaccine uptake or coverage, including any associated economic analyses.

In addition, we included primary studies identified from searching the references from other review articles identified in the search that fitted inclusion criteria.

### Study selection process

One reviewer screened articles by title and manually de-duplicated records. Two reviewers screened potentially relevant abstracts independently. Any disagreement was resolved by discussion, based on the inclusion criteria. Three reviewers agreed the final inclusions.

## Results

**Fig. 2:**
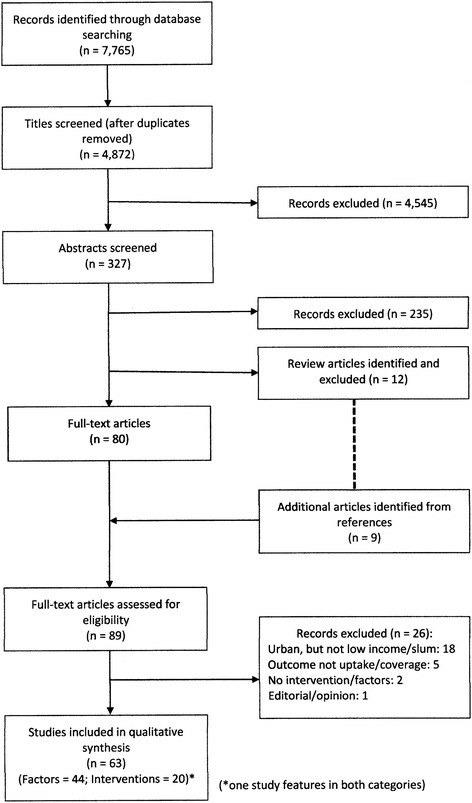
PRISMA flowchart of literature selection

Of 4872 unique articles, 327 abstracts were screened, leading to 80 full text articles being reviewed, along with nine additional studies identified from the references of 12 review articles [6, 26–36]. In total 63 studies were included (Fig. 2). Forty-four studies looked at factors associated with immunization coverage and 20 studies evaluated interventions. One study appears in both categories.

### Factors associated with immunization coverage

The qualitative synthesis of factors identified from 44 studies is presented below, categorized into 4 groups: socio-economic characteristics; migration status; information, beliefs and behavior; and health services. All studies were cross-sectional surveys, aside from one qualitative study [37], and one ecological study [38]. Quantitative synthesis of measures of effect was not able to be performed due to heterogeneity in study design, population and methods.

Studies were conducted in populations in India (*n* = 23) [7, 9, 38–58], Pakistan (*n* = 3) [59–61], Iran (*n* = 1) [62], China (*n* = 1) [63], Kenya (*n* = 6) [10, 64–68], Nigeria (*n* = 4) [8, 69–71], Burkina Faso (*n* = 1) [10], Ethiopia (*n* = 1) [72], Democratic Republic of Congo (DRC)(*n* = 1) [37], Central African Republic (CAR)(*n* = 1) [73], Zambia (*n* = 1) [74], and Brazil (*n* = 2) [75, 76],

#### Socio-economic and demographic characteristics

Socio-economic (SE) status was measured in a variety of ways, including using measures of wealth or income, employment status, education, or surrogate markers like receipt of government payments.

In India, the impact of SE factors has been studied extensively in low-income urban and slum areas. An ecological study using data from the Indian National Family Health Survey showed that the children in the lowest wealth quartile in urban areas have significantly lower coverage (around 40%) than in any other urban wealth group [38]. Another study examined the survey data in more detail to compare a wider range of SE factors and outcomes of 1877 children in less developed Empowered Action Group states with more developed northern counterparts and found that having an illiterate mother or father, poverty, and being a 3rd born child or greater had the greatest effect on immunization coverage [46]. However, studies conducted in individual locations paint a more heterogeneous picture. Mothers’ education was associated with lower coverage in some studies [39, 49, 51], but not in others [45, 53]. Similarly, female children had lower coverage in some studies [39, 45, 51], but no gender difference was found in others [9, 49, 50]. In one study malnutrition was also found to be much more common in unimmunized slum-dwelling children [44].

Elsewhere, a study in a slum in Karachi, Pakistan, found lower coverage in lower SE groups [59]. However, the study reported a much more significant effect of being from a marginalized ethnic group, particularly if associated with illegal or insecure residential status. Another study in Pakistan found mothers’ education was a significant predictor of measles containing vaccine (MCV) coverage in urban areas [60]. However, the impact of other SE factors was different in different localities.

The findings from sub-Saharan African countries are similar in their diversity. Across 3 slum populations in Kenya factors associated with immunization coverage included: maternal education, employment and age; child’s birth order; number of children, place of birth; and household assets and expenditure [64–66]. Ethnic group was a significant predictor of MCV uptake in Nairobi [67]. A paired study conducted in slums in Nairobi and Ouagadougou found that while the SE factors in each slum were similar, children in Nairobi were 11.5 times more likely to be unvaccinated, suggesting a powerful environmental effect [10]. In Jigjiga, Ethiopia, maternal age and literacy, place of residence, tetanus immunization status, place of delivery and household visit by health workers were the most important predictors of completing immunization [72].

In Sao Palo, Brazil, a study of 258 children in a philanthropic day care centre found premature birth, malnourishment, inadequate housing and poor prenatal care to be associated with lower coverage [75]. However, a larger Brazilian study involving 17,000 children found that those in lower SE groups had higher immunization rates than their wealthier peers [76]. Although this association is not fully explained in the paper, the authors found that parents in higher socioeconomic quintiles had higher vaccine refusal rates and hypothesize that progressive reduction in VPD incidence over many years may have led to complacency, alongside unfounded vaccine safety concerns widely reported in other countries.

#### Migration status

Four studies demonstrated a negative effect of migration status on immunization coverage in India [7, 53], China [63], and Nigeria [71]. In India, recent migrants were found to have lower coverage than settled migrants (living in new urban location for >12 months), which then resolved to the slum-area average over time [53]. A study comparing reasons for under-immunization given by migrant and non-migratory parents in an Indian slum cited mother or both parents being too busy; parent returned to home village; parent unaware of place or time of immunization; and lack of awareness for the need for immunization as the main reasons for under-immunization [7]. In Nigeria, children of urban non-migrant mothers had 67% higher chance of being fully immunized than migrant children, which was attributed to the disrupting force of migration [71]. However, other characteristics, including: children being of higher birth order; being a mother aged <18 years; and lower SE status, also played a significant role, independent of migration status after regression modelling. Thus, there are elements of similarity of the risks of low immunization coverage as faced by all urban poor communities, but these are exacerbated by the disruptive force of migration.

#### Information, beliefs and behavior

Many studies that collected information on SE characteristics also asked parents why their children were not fully vaccinated. In India, frequently identified reasons included: unaware of the need for vaccines; [7, 9, 39, 40, 42, 45, 47, 49, 50, 52, 54, 56, 57] parents being too busy; [7, 9, 39, 42, 54, 55] traveled to place of origin; [7, 39, 54–56] and unaware of clinic location or timing [7, 9, 42, 47, 52, 54].

Unfortunately, there are fewer studies available from other countries to make similar comparisons. In two studies conducted in Pakistan, maternal knowledge of immunization was an important factor; [61] and the reasons for under-immunization given by mothers were to do with ‘carelessness’ or difficulty in accessing services [60]. In Ibadan, Nigeria, a study found fear of side-effects, maternal awareness, and parents being too busy to attend clinic to be significant; [8] in neonates in Benin City, Nigeria, a study found that SE and education status were associated with delayed immunization; [69] and the 3 main reasons for under-vaccination identified in a study from Bangui, Central African Republic, were the mother being too busy, negative attitude of health workers, and lack of access to information [73].

One qualitative study conducted in a slum in DRC investigated health service access using focus group interviews [37]. It painted a detailed picture of parents conflicting views and beliefs, including concerns over out-of-pocket expenditure and being suspicious of free services (including vaccines), while seeing some services as beneficial, but not acceptable due to lack of information and distrust in the government.

#### Health services

A study that used mapping techniques to evaluate health service access in Agra, India, found that the presence of a health center within 2 km of the slum doubled the chances of a child being completely immunized [51]. A similar study in Lusaka, Zambia, showed that further distance from service points were associated with lower coverage [74]. Timing of services is also important, where offering services only on one particular day is associated with reduced coverage in neonates in Nigeria [69]. However, slum-dwelling populations may also be less likely to access health services due to fear of costs, risk of lost income, or lack of local knowledge [48]. A study from Nigeria showed that even when services are provided free, urban dwellers consume less than their rural counterparts [70]. However, another study found a high level of payment for services that should have been provided for free, including immunization and pre-natal care. This may be a common experience in LMICs, due to low payments made to health workers, and when present, may affect the long-term effectiveness of vaccine programmes. However, when people do access healthcare higher patient satisfaction and provision of accurate information was shown to lead to increased attendance for repeat vaccine doses in an Indian slum community [58]. Accessing pre-natal care was shown to have a positive impact on immunization coverage in India [41], Ethiopia [72], and Brazil [75].

A missed vaccination opportunity is when health workers interact with a child who is under-immunized and could have been offered a vaccination, but for some reason do not [27]. A study conducted in 6 health facilities in a Nairobi slum found that vaccine coverage could have been increased if all missed vaccination opportunities had been taken [68].

### Interventions

Twenty studies were identified that looked at interventions to increase immunization uptake and are displayed in Table 3. These were conducted in India (*n* = 5) [77–81], Pakistan (*n* = 5) [82–86], Bangladesh (*n* = 3) [87–89], Zambia (*n* = 2) [74, 90], Uganda (*n* = 1) [91], Kenya (*n* = 1) [92], Brazil (*n* = 1) [93], Guatemala (*n* = 1) [94], and Mongolia (*n* = 1) [95].

**Table 3:** Showing the results of studies examining interventions to increase immunization uptake in urban poor and slum communities in low and middle-income countries

Author, Year [Reference]	Country (Area)	Intervention Category	Intervention	Study design	Study population	Sample size & comparison	Outcome	Comments
Uddin, 2010 [88]	Bangladesh (Dhaka)	Multi-C	Extended hours, provider training, screening tool, community group.	Before (bf) and after (aft)	Children 12–23 months in a Dhaka slum.	529 before, 526 after.	Fully immsd increased from 43% to 99% (*p* < 0.000)	Increases seen across range of individual vaccines, and in both children of working and non-working mothers.
Hayford, 2014 [87]	“	“	“	Economic evaluation	“	-	Cost of $20.95 per fully immsd child	Total cost for intervention for 1 year $18,300.
Pradhan, 2012 [81]	India (Patna)	Multi-C	Outreach services, additional staff, task shifting, link workers, geographic monitoring, community involvement, additional supervision.	Before and after	All eligible living in slums in Patna	Estimated eligible population immsd before and after intervention.	BCG 29% bf, 64% aft DPT1 28% bf, 62% aft DPT3 21% bf, 49% aft MCV 23% bf, 51% aft TT1 15% bf, 22% aft TT2 10% bf, 28% aft	Increases observed across a range of vaccines in a population of approx. 25,000 children, however statistical significance not reported.
Agarwal, 2008 [77]	India (Indore)	Multi-C	Increasing awareness & demand, improve vaccine supply and accessibility, community links.	Before and after	Children aged 12–23 months living in 79 slum areas	Eligible children within estimated 150,000 study population.	Fully immsd increased from 32% to 72% between 2003 (bf) and 2006 (aft).	Primarily descriptive, with limited detail on outcomes achieved and no statistical analysis.
Khan, 2006 [85]	Pakistan (Karachi)	Multi-C	Information, education, community involvement, intensive vaccination campaign.	Cluster RCT	Children aged 2 to 16 years in squatter settlements.	21,059 children in 60 clusters.	Achieved 74% coverage in previously unvaccinated population.	Purpose of study was to test effectiveness of typhoid polysaccharide vaccine, using Hep A as a control, but also reported on campaign design.
Poulos, 2004 [96]	India (New Delhi)	Multi-C	Mass vaccination vs. school campaign vs. targeted campaign at 2–5 year old children.	Economic evaluation	All people within a slum area in new Delhi	26 clusters	Assuming cost of $1 per typhoid vaccine: Mass campaign = $50 per case avoided, School campaign = $41, and targeted to young children = $14.	Examination of the economic benefits of typhoid vaccine campaigns from a societal perspective using different methods in a slum area.
Mbabazi, 2012 [92]	Kenya (Nairobi and Nyanza/Western Provinces)	Multi-C	House to house canvassing, community mobilization, mobile phone documentation, web application monitoring.	Cross-sectional (post-hoc data only)	Children in high-density urban poor communities.	164,643 households, with 161, 695 children	Post campaign monitoring found measles coverage of 96% reported (92% confirmed).	Additional strategy as part of a mass measles campaign to increase coverage of the campaign. 75% households reported acceptance of supplemental measles vaccination prior to intervention.
Lhamsuren, 2012 [95]	Mongolia (Ulaanbaatar)	Multi-C	Reaching Every District (RED) strategy [104].	Cross-sectional & qualitative.	At risk children in urban poor communities, focusing on one district of 22,726 people)	3126 at risk children under 15 years old in selected community.	Immsd an additional 477 at risk children (15% of total number of eligible children).	Detail provided on barriers to imms services. Cost in study district = $14,166, which also included other maternal and child health interventions.
Igarashi, 2010 [74]	Zambia (Lusaka)	Outreach	Growth Monitoring Plus (GMP): outreach vaccination and other child health services into under-served slum areas using community volunteers.	Interrupted time series	Children in four slum area, split into 2 primary areas and 2 areas with 2-year time-lag.	1128 (584 in primary site, 544 in time-lag intervention site)	Full imms coverage increased from 52.6% at baseline to 68.8% at final measurement in primary area (*p* < 0.001) and 43.1% to 56.7% in lag area (non-significant).	Length of residence was significantly longer in primary area, which may explain the differential effect. Frequency of attendance at GMP+ services significantly associated with higher coverage (OR 1.27, *P* < 0.001)
Sasaki, 2011 [90]	“	“	“	Before and after cross-sectional surveys	Children in one slum area of 48.798 people (one of the 4 areas included in the above study).	280 sampled households.	DPT3 increased 75.7% (bf) to 87.3% (aft) and measles from 66.8% (bf) to 76.1% (aft).	Closer distances to imms service points significantly associated with higher coverage, and impact of this reduced with GMP+ outreach services.
Ansari, 2010 [78]	India (Aligarh City)	Outreach	Immunisation outreach camps	Cross-sectional	Children <5 in slum areas of Aligarh City	2531	13,989 vaccines administered to the 2531 children attending services.	Population coverage not reported. Significantly lower imms coverage observed in female children attending.
Prabhakaran, 2014 [80]	India (New Delhi)	Outreach	Mobile health clinic	Economic Evaluation	Children <5 years living in Dakshinpuri extension resettlement colony.	1583 children attending the clinic in 1 year.	1583 children received 8488 vaccinations through the service at a cost of 66.14 Rupees per vaccine (US$0.10)	Imms services provided alongside a range of other health services.
Uddin, 2016 [89]	Bangladesh (Dhaka)	Reminder/ recall	SMS (text) message reminders managed using mTika software system.	Before and after	Both children aged 0–11 months in hard-to-reach rural areas and urban street children in slums.	2823 urban street children (intervention 518 bf and 520 aft, with 1785 controls)	Urban intervention: 40.7% (bf) to 57.1% (aft) compared to controls 44.5% (bf) to 33.9% (aft).	Adjusted OR = 3.0 (95% CI 1.4–6.4). Decline in control population imms coverage also noted in rural population (not shown here).
Kazi, 2014 [83]	Pakistan (Karachi)	Reminder/ recall	SMS (text) message monitoring of immunisation activities.	Cross-sectional	20 clusters of 200–250 households in 3 high polio risk areas.	28 households with children under 5 in each cluster.	Coverage in population who replied to messages was 74.5% (95% CI 71.6–77.4) which was very similar to result found using WHO lot quality assurance sampling.	Text messages sent to parents to confirm if immunization staff had visited and vaccinated child. If no answer, follow-up phone calls made.
Domek, 2016 [94]	Guatemala (Guatemala City)	Reminder/ recall	SMS (text) message reminders	RCT pilot	Children aged 8–14 weeks presenting at a clinic serving a low-income population.	321 (160 intervention, 161 control)	Higher proportion in intervention group completed series (84.4% vs 80.7%), which was not statistically significant.	Three reminders sent to intervention parents. Loss to follow-up 25 in intervention and 31 in control groups. Demonstrated feasibility.
Mukanga, 2005 [91]	Uganda (Mulago II Parish, outside Kampala)	Reminder/ recall	Child health cards	Cross-sectional	Children aged 0–24 months	260 households	66% children had child health cards, and were almost 10 times more likely to be fully immsd than those without (OR = 9.55, 95% CI 3.19–29.45)	Children born in a health facility were 4 times more likely to have a health card than those born at home.
Owais, 2011 [84]	Pakistan (Karachi)	Education	Education session delivered by community health workers using pictorial message.	RCT	Mothers of children sampled from 5 low-income areas of Karachi	366 mother-infant pairs (179 in intervention, 178 in control group finally snslysed)	Increase in DPT3/Hep B coverage of 39% in intervention group (RR = 1.39, 95% CI: 1.06–1.81)	27% (312) of sampled mothers declined to participate.
Anjum, 2004 [86]	Pakistan (Karachi)	Education	Education messages provided to mothers by medical students	Randomized, controlled before and after	People living in Sikanderabad squatter settlement in Karachi.	317 households (110 intervention 207 controls)	Follow-up 4 years post intervention found significant increase in full imms intervention area (46.5% bf, 75% aft, *p* < 0.005) and no sig difference in control area. However, no test for interaction performed, compromising the statistical results.	The results are limited by poor study design. Although uptake of imms services significantly increased in both intervention and control households the groups were poorly matched with very divergent baseline service use, preventing firm conclusions being drawn about intervention effectiveness.
Shei, 2014 [93]	Brazil (Salvador)	Incentives	Conditional cash transfer (Bolsa Familia) to people on low-incomes.	Nested cross-sectional survey within prospective cohort	Bolsa Familia recipients in a slum area of 14,000 people.	1266 children from 3000 randomly sampled households (841 beneficiaries, 425 controls)	Recipient children under 7 years 2.8 times more likely to attend services for vaccination (OR 2.8, 95% CI 1.4–5.4, *P* = 0.002).	Also increased odds of attending for growth monitoring (OR 3.1, *P* < 0.001) and health checks (OR = 1.6, *p* = 0.061). No impact on children older than 7.
Chandir, 2010 [82]	Pakistan (Karachi)	Incentives	Food/medicine coupon incentives worth US$2 for follow-up DTP vaccinations.	Quasi-experimental with non-simultaneous controls	11 sub-districts, including middle and very-low income households.	2561 intervention, 2051 controls.	Completion of DTP 3 vaccinations higher in intervention group (Adjusted RR 2.2, 95% CI 1.95–2.48, *p* < 0.001)	Significant loss in the control cohort, only 847 (41% enrolled) entering final analysis.

*Multi-C* multi-component intervention, *Bf* before, *Aft* after, *Imms/Immsd* immunize/immunized, *BCG* Bacillus Calmette-Guérin vaccine, *DPT1* first Diphtheria, Pertussis & Tetanus vaccine, *DPT3* third Diphtheria, Pertussis & Tetanus vaccine *MCV* meningococcal group C vaccine, *TT1/2* first/s tetanus toxoid vaccine, *GMP* Growth Monitoring Plus programe, *Hep B* Hepatitis B vaccination, *OR* Odds Ratio, *RR* Risk ratio, *95% CI* 95% Confidence Interval

We have divided them into five categories: multi-component interventions (*n* = 8); outreach programs (*n* = 4); reminder/recall systems (*n* = 4); education (*n* = 2); and those considering incentives (*n* = 2)

#### Multi-component interventions

We found evidence of effectiveness for interventions involving multiple components designed to meet the specific needs of a slum-dwelling community, especially if they have been designed and delivered with community involvement.

Two related studies reported on an intervention conducted in Dhaka, Bangladesh. The first is a non-controlled before-and-after evaluation of the intervention comprising four components: extended services hours; provider training; a screening tool; and a community support group [88]. Analysis of 529 children before and 526 after the intervention showed increased coverage over 12 months, with complete immunization rising from 43% to 99%. The second study calculated the cost of the programme at $20.95 per fully immunized child [87]. A similar programme in low-income areas of Patna, India, involving enhanced service delivery (outreach; additional nursing staff; task-shifting; geographic monitoring; community mobilization; supervision and communication components) was evaluated using before-and-after analysis of routinely collected data, and showed increases in immunization uptake e.g. DTP3 increased from 21% to 49%, MCV from 23% to 51% [81]. Another study evaluated the Urban Health Programme (combining demand and supply side interventions with increased clinic accessibility and community involvement) in Indore, India, which showed an increase in complete immunization among infants from 32% to 72% [77].

Two studies considered typhoid vaccination specifically. A cluster RCT involving 21,059 children was conducted in squatter communities in Karachi, Pakistan, to evaluate a campaign to increase typhoid vaccine coverage (involving information provision; community involvement; increased services; and staff training), which achieved 74% coverage in the target population, with maintenance of cold-chain [85]. A study in New Delhi, India, undertook economic analyses of different vaccination campaigns against typhoid and concluded that a targeted program for pre-school children was most cost-effective (US$14 per case averted), when compared to a school campaign (US$41) or general mass campaign (US$50) [96].

A large study (*n* = 161,695 children) in urban Nairobi and Nyanza provinces focusing on populations living in low-income, high-density environments demonstrated how the use of community volunteers calling door-to-door and providing standardized information using mobile phone technology can contribute to achieving high coverage (92%) during a planned mass MCV campaign [92].

A study conducted in Ulaanbaatar City, Mongolia, reports a before-and-after analysis following the implementation of the ‘Reaching Every District’ (RED) strategy by the ministry of health in an urban poor community, which reached an additional 477 children at risk of under-immunization, 15% of the total eligible population in the entire district, and also improved organizational structures and staff motivation [95].

#### Outreach programmes

Two related studies reported on the Growth Monitoring Program Plus programme, which involved providing monthly outreach services implemented in peri-urban areas of Lusaka, Zambia [74, 90]. Comparing early implementation sites with late implementers, immunization coverage was shown to significantly increase in both areas, from 52.6% to 68.8% (early) and 43.1% to 56.7% (late), even after controlling for socio-economic characteristics.

A study on an outreach services provided in Aligarh City, India, as part of a campaign reported success in immunizing over 2500 children in a low-income district and improving overall complete immunization coverage, demonstrating both feasibility and acceptability [78]. Another study in India provided descriptive evidence for the feasibility of providing vaccinations through a mobile health clinic in slum communities in Delhi, at the cost of RS66.14 per vaccine delivered (US$0.10) [80].

#### Reminder/recall

Three studies have shown varying levels of success in the use of text message (SMS) systems. In Bangladesh, the use of SMS reminder system in both urban street dwellers and a rural ‘hard-to-reach’ population was evaluated [89]. The results showed an increase in coverage in both populations, with an adjusted odds ratio of complete immunization of 3.0 (95% confidence interval (CI): 1.4–6.4) among the urban children. In Pakistan, an SMS system was successfully used to monitor provision of polio immunization by asking urban community members to report whether their children had been immunized by vaccinators [83]. A proof-of-concept study in an urban poor community in Guatemala City showed that an SMS system to remind mothers to receive pentavalent vaccine was feasible and acceptable, but found no difference in vaccine uptake [94].

A study in a slum community in Uganda showed that children born at a health facility, or whose mother was unwell during the pregnancy were more likely to have an immunization reminder card, and that these children were 10 times more likely to be up to date for all immunizations when compared to children without cards [91].

#### Education

Two studies considered the effect of an intervention focussing on education or information provision to parents in Pakistan. An RCT conducted in Karachi involved community health workers delivering pictorial messages to encourage immunization [84]. The intervention resulted in a 39% increase (OR 1.39, 95% CI: 1.06–1.81) of DTP3/Hepatitis B completion, although 27% of sampled women declined to take part. A randomized controlled before-and-after study of the provision of education sessions by medical students to mothers in a squatter settlement found an increase in complete immunization in the intervention group [86]. However, health centre utilisation for vaccination in intervention and control groups was significantly different at baseline (30% vs 13% respectively), suggesting participants were poorly matched. Additionally, this study was excluded from a related Cochrane Review due to its poor study design [26], and thus provides only weak evidence for this intervention.

#### Incentives

Two studies considered the effect of incentives on increasing vaccination uptake. A study of the Bolsa Familia program of conditional cash transfers in slums in Salvador City, Brazil, found that recipient families had higher use of health clinics and increased odds of vaccination uptake (OR 2.8; 95% CI: 1.4–5.2) [93]. A study conducted in Pakistan involving nearly 4000 children investigated the effect of providing a coupon redeemable for food or medicine (worth US$2) to incentivize attendance amongst low-income mothers in Karachi at follow-up immunization appointments [82]. The results showed increase (OR 2.2, 95% CI: 1.95–2.48) in timely completion of the DTP immunization series

## Discussion

A wide range of socio-economic and behavioural factors have been associated with immunization status in different slum and urban poor contexts. While some of this diversity may result from differences in study methods, much may also be from true differences in the factors in the underlying populations. One included study from Pakistan applied the same methods in multiple settings simultaneously and reached the conclusion that *‘one size does not fit all’,* which is supported by the evidence in this review [60].

Further qualitative work is required to better understand the interlinking socioeconomic and demographic factors that influence immunisation coverage inequalities identified through analysis of surveys or population level data sources. The application of intersectionality theory, a method of systematically considering the multiple social labels individuals hold (e.g. ethnic group, gender, social class, migration status), may also provide additional useful detail in slum populations [97].

### Improving immunization coverage

The evidence presented here demonstrates the relatively small number of interventional studies that have been conducted in slum populations. In addition, several of the studies have significant methodological flaws, requiring caution in the interpretation of the results. However, when comparing the evidence base identified here with the wider literature, it is notable that no interventions consider the importance of providing services to new migrants from rural areas in slum communities and that community involvement may be especially useful when designing interventions in slum areas.

A Cochrane review of interventions to improve coverage of childhood immunisation in low and middle income countries in all populations was published in 2014 also found a limited evidence base [26]. Of the studies described here, only one evaluating a maternal education programme was also included in the Cochrane review [84]. However, a comparison of the effectiveness for the type of interventions identified in this paper and in the Cochrane review are presented in Table 4.

**Table 4:** A comparison of results for evidence of effectiveness of interventions to increase vaccination coverage in LMICs from a Cochrane review, [26] and in slum populations identified in this paper

Intervention type	Strength of evidence of benefit	
	Cochrane Review (general populations)	Slum populations
Education	Moderate	Some evidence of potential benefit
Education and reminder cards	Low	Not tested
Household financial incentive	No effect	Some evidence of potential benefit
Outreach and financial incentives	Low	Not tested
Home visits	Low	Evidence of benefit, where distance is significant.
Integrating immunisation with other health services	Low	Not tested
Text messaging	Not tested	Some evidence of potential benefit
Community involvement	Not tested	Important factor in effective studies.

Multi-component interventions that tackle multiple factors that can contribute to low coverage have some evidence for effectiveness [77, 81, 85, 88, 92, 95]. An important component was community involvement, which enables the individual context of a slum to be considered, although this was not identified separately in the Cochrane review. This should include consideration of minority ethnic groups, who may suffer from lower coverage [59, 67]. This matches similar research conducted in deprived urban communities in high-income countries [98].

The outreach programs identified in the literature focused specifically on reducing physical distance between communities and health services have evidence of effectiveness [74, 78, 80, 90]. This finding matches that of a recent systematic review looking at improving immunization in LMIC urban areas generally, which concluded that outreach visits worked well in densely populated areas [99]. However, overall the authors identified fewer papers than are included in this study despite having a broader topic and longer time-frame. Outreach may be particularly relevant for slum communities, where there may be a lower density of health facilities, and should be a focus of further research.

No interventions were identified that reduced the social distance that some slum residents may experience when accessing health services. Specifically, no interventions were identified that provided services to migrants from rural areas, despite that migration was identified as having a universally negative effect on immunization coverage. Slums are often the first entry point of new migrants to an urban area [23], and the process of migration leads to an understandable disruption in parents’ ability to access health services, leading to lower coverage [53, 71].

Reminder/recall systems have good evidence for effectiveness in high-income countries and are considered as a core component of any immunization programme [100]. However, the provision of reminder/recall systems in LMICs is challenging, especially in slums, due to informal road systems, lack of addresses and limited access to electronic communications. However, of the three studies presented here, two show that SMS reminders can be effective in a slum context, [83, 89] and the other showed feasibility as part of a pilot RCT, although found no difference in coverage [94].

Of the two studies evaluating education programmes identified here, one showed some evidence of effectiveness [84], but the other was compromised by methodological limitations [86]. However, community-based education programmes have evidence for effectiveness in increasing immunization uptake in LMICs generally and warrant further investigation in slum contexts [26]. The use of incentives is not supported by evidence from a recent Cochrane review of interventions to improve immunization in LMICs generally, unless incentives are combined with outreach [26]. However, both studies identified here had positive effects that also warrant further investigation [82, 93].

### Limitations

The quality of the included studies was only poor to medium, with no high quality randomised trials, making assessment for risk of bias challenging. We included studies conducted in slums alongside other urban poor communities, which may not be commensurate. Few studies reported negative results, suggesting publication bias overall. Several studies reported in Chinese were excluded. No evidence was identified for many countries with significant slum-dwelling populations, such as Indonesia, the Philippines, Sudan, Mozambique and Madagascar.

## Conclusions

Different factors affect immunization coverage in different urban poor and slum contexts. Immunization services should be designed and provided to slum-dwelling communities in consultation with the people living there, considering the local context and avoiding constructing barriers to access, such as geographic and social distance, cost and timing. Interventions should be designed and tested to increase immunization in new migrants from rural areas.

## Additional file

Additional file 1: Search strategy. Search strategy. Description of data: search strategy used in Medline. (DOCX 18 kb)

## Abbreviations

CAR: Central African Republic
DRC: Democratic Republic of Congo
DTP3: Diphtheria, Tetanus, Pertussis vaccine, 3rd dose
LMIC: Low and Middle Income Countries
MCV: Measles Containing Vaccine
RCT: Randomized Controlled Trial
SE: Socio-economic
SMS: Short Message Service, text message
VPD: Vaccine Preventable Disease
