# Supplementary material for: Immunization, urbanization and slums – a systematic review of factors and interventions
Source: BMC Public Health. 2017 Jun 8;17:556. doi: 10.1186/s12889-017-4473-7 (PMC5465583; doi:10.1186/s12889-017-4473-7)
Supplement: Additional file 1: — Search strategy. Search strategy. Description of data: search strategy used in Medline. (DOCX 18 kb) [file 12889_2017_4473_MOESM1_ESM.docx]

**Additional File 1: Search Strategy**

1 developing country/ (67333)

2 ((developing or less* developed or under developed or underdeveloped or middle income or low* income or underserved or under served or deprived or poor*) adj (economy or economies)).ti,ab. (364)

3 ((developing or less* developed or under developed or underdeveloped or middle income or low* income or underserved or under served or deprived or poor*) adj (countr* or nation? or population? or world)).ti,ab. (62376)

4 (low* adj (gdp or gnp or gross domestic or gross national)).ti,ab. (178)

5 (low adj3 middle adj3 countr*).ti,ab. (5349)

6 (lmic or lmics or third world or lami countr*).ti,ab. (3749)

7 transitional countr*.ti,ab. (116)

8 or/1-7 (108368)

9 Cambodia/ (2572)

10 (cambodia or khmer republic).mp. (3282)

11 North Korea/ (149)

12 (north korea or (democratic people* republic adj2 korea)).mp. (311)

13 Myanmar/ (1587)

14 (myanmar or burma).mp. (2534)

15 or/9-14 (5927)

16 Tajikistan/ (662)

17 (tajikistan or tadzhik or tadzhikistan or tajikistan).mp. (807)

18 or/16-17 (807)

19 Haiti/ (2638)

20 Haiti.mp. (3137)

21 or/19-20 (3137)

22 Afghanistan/ (2651)

23 Afghanistan.mp. (4688)

24 Bangladesh/ (8201)

25 Bangladesh.mp. (10195)

26 Nepal/ (5861)

27 Nepal.mp. (6997)

28 or/22-27 (21371)

29 Benin/ (1217)

30 (Benin or Dahomey).mp. (2868)

31 Burkina Faso/ (2582)

32 (Burkina Faso or Burkina Fasso or Upper Volta).mp. (3261)

33 Burundi/ (565)

34 Burundi.mp. (753)

35 Central African Republic/ (668)

36 (Central African Republic or Ubangi-Shari).mp. (936)

37 Chad/ (603)

38 Chad.mp. (957)

39 Comoros/ (240)

40 (Comoros or Comoro Islands or Mayotte or Iles Comores).mp. (440)

41 Democratic Republic Congo/ (0)

42 ((democratic republic adj2 congo) or belgian congo or zaire).mp. (3236)

43 Eritrea/ (245)

44 Eritrea.mp. (387)

45 Ethiopia/ (8463)

46 Ethiopia.mp. (9608)

47 Gambia/ (2211)

48 Gambia.mp. (2726)

49 Guinea/ (765)

50 (Guinea not (New Guinea or Guinea Pig* or Guinea Fowl)).mp. (3056)

51 Guinea-Bissau/ (807)

52 (Guinea-Bissau or Portuguese Guinea).mp. (1000)

53 Kenya/ (12876)

54 Kenya.mp. (15567)

55 Liberia/ (933)

56 Liberia.mp. (1276)

57 Madagascar/ (2744)

58 (Madagascar or Malagasy Republic).mp. (3789)

59 Malawi/ (3896)

60 (Malawi or Nyasaland).mp. (4852)

61 Mali/ (1957)

62 Mali.mp. (2934)

63 Mozambique/ (1733)

64 (Mozambique or Portuguese East Africa).mp. (2483)

65 Niger/ (998)

66 (Niger not (Aspergillus or Peptococcus or Schizothorax or Cruciferae or Gobius or Lasius or Agelastes or

Melanosuchus or radish or Parastromateus or Orius or Apergillus or Parastromateus or Stomoxys)).mp. (2422)

67 Rwanda/ (1750)

68 (Rwanda or Ruanda).mp. (2227)

69 Sierra Leone/ (1088)

70 Sierra Leone.mp. (1494)

71 Somalia/ (1271)

72 Somalia.mp. (1652)

73 Tanzania/ (9002)

74 (Tanzania or Zanzibar).mp. (10634)

75 Togo/ (936)

76 (Togo or Togolese Republic).mp. (1242)

77 Uganda/ (9422)

78 Uganda.mp. (11228)

79 Zimbabwe/ (5089)

80 (Zimbabwe or Rhodesia).mp. (6156)

81 or/29-80 (86235)

82 Indonesia/ (8068)

83 indonesia.mp. (10562)

84 kiribati/ (1033)

85 Kiribati.mp. (119)

86 "Federated States of Micronesia"/ (0)

87 micronesia.mp. (1347)

88 Laos/ (1486)

89 (laos or (lao adj2 democratic republic)).mp. (1995)

90 marshall islands/ (1033)

91 marshall island*.mp. (247)

92 Mongolia/ (1394)

93 (mongolia or mongolian people* republic).mp. (2883)

94 Papua New Guinea/ (3061)

95 Papua New Guinea.mp. (4449)

96 Philippines/ (7185)

97 Philippines.mp. (9228)

98 Samoa/ (269)

99 samoa.mp. (885)

100 solomon islands/ (944)

101 Solomon Islands.mp. (559)

102 Melanesia/ (944)

103 melanesia.mp. (1257)

104 Timor-Leste/ (112)

105 (Timor-Leste or East Timor).mp. (318)

106 Vanuatu/ (294)

107 Vanuatu.mp. (513)

108 Viet Nam/ (9881)

109 (Viet Nam or Vietnam).mp. (13440)

110 or/82-109 (43624)

111 Armenia/ (1263)

112 Armenia.mp. (1497)

113 "Georgia (republic)"/ (1483)

114 (georgia not (georgia adj3 state)).mp. (14052)

115 "yugoslavia (pre-1992)"/ or yugoslavia/ (9060)

116 Kosovo/ (74)

117 kosovo.mp. (595)

118 Kyrgyzstan/ (1185)

119 (kyrgyzstan or kyrgyz republic or kirghizia or kirghiz).mp. (1382)

120 Moldova/ (624)

121 Moldova.mp. (824)

122 Ukraine/ (15158)

123 Ukraine.mp. (16046)

124 Uzbekistan/ (1861)

125 Uzbekistan.mp. (2073)

126 or/111-125 (44518)

127 Bolivia/ (2105)

128 Bolivia.mp. (2949)

129 El Salvador/ (770)

130 salvador.mp. (2599)

131 Guatemala/ (2537)

132 Guatemala.mp. (3311)

133 Guyana/ (589)

134 Guyana.mp. (942)

135 Honduras/ (949)

136 Honduras.mp. (1442)

137 Nicaragua/ (1237)

138 Nicaragua.mp. (1609)

139 Paraguay/ (632)

140 Paraguay.mp. (1132)

141 or/127-140 (12755)

142 Djibouti/ (199)

143 (Djibouti or French Somaliland).mp. (323)

144 Egypt/ (12886)

145 Egypt.mp. (15468)

146 Morocco/ (4542)

147 Morocco.mp. (5620)

148 Syrian Arab Republic/ (0)

149 (Syria or Syrian Arab Republic).mp. (1694)

150 Gaza.mp. (828)

151 Yemen/ (1138)

152 Yemen.mp. (1509)

153 or/142-152 (24839)

154 Bhutan/ (256)

155 Bhutan.mp. (391)

156 India/ (85475)

157 India.mp. (101029)

158 Pakistan/ (13150)

159 Pakistan.mp. (15589)

160 Sri Lanka/ (4734)

161 (Sri Lanka or Ceylon).mp. (5938)

162 or/154-161 (120058)

163 Cameroon/ (4289)

164 Cameroon.mp. (5460)

165 Cape Verde/ (127)

166 (Cape Verde or Cabo Verde).mp. (427)

167 Congo/ (1589)

168 (congo not ((democratic republic adj3 congo) or congo red or crimean-congo)).mp. (2099)

169 Cote d'Ivoire/ (2703)

170 (Cote d'Ivoire or Ivory Coast).mp. (3542)

171 Ghana/ (5708)

172 (Ghana or Gold Coast).mp. (7032)

173 Lesotho/ (328)

174 (Lesotho or Basutoland).mp. (506)

175 Mauritania/ (361)

176 Mauritania.mp. (524)

177 Nigeria/ (24233)

178 Nigeria.mp. (26807)

179 "sao tome and principe"/ (714)

180 (sao tome adj2 principe).mp. (98)

181 Senegal/ (5019)

182 Senegal.mp. (6274)

183 Sudan/ (4206)

184 Sudan.mp. (7176)

185 Swaziland/ (410)

186 Swaziland.mp. (608)

187 Zambia/ (3544)

188 (Zambia or Northern Rhodesia).mp. (4389)

189 or/163-188 (62681)

190 American Samoa/ (155)

191 american samoa.mp. (311)

192 China/ (115640)

193 (china or (chinese adj3 republic)).mp. (143850)

194 Fiji/ (753)

195 fiji.mp. (1194)

196 Malaysia/ (12029)

197 malaysia.mp. (14457)

198 marshall islands/ (1033)

199 marshall islands.mp. (231)

200 Palau/ (172)

201 Palau.mp. (307)

202 Thailand/ (22143)

203 (Thailand or Siam).mp. (27681)

204 tuvalu/ (1033)

205 Tuvalu.mp. (48)

206 or/190-205 (185288)

207 Albania/ (711)

208 Albania.mp. (939)

209 Azerbaijan/ (1122)

210 Azerbaijan.mp. (1413)

211 Belarus/ (2007)

212 (belarus or byelarus or belorussia).mp. (2442)

213 "Bosnia and Herzegovina"/ (1749)

214 bosnia.mp. (2283)

215 Bulgaria/ (5943)

216 Bulgaria.mp. (6926)

217 Hungary/ (17115)

218 hungary.mp. (19919)

219 Kazakhstan/ (2279)

220 (Kazakhstan or kazakh).mp. (2861)

221 "Macedonia (republic)"/ (442)

222 Macedonia.mp. (870)

223 "Montenegro (republic)"/ (0)

224 Montenegro.mp. (603)

225 Romania/ (9045)

226 Romania.mp. (10326)

227 Serbia/ (2128)

228 serbia.mp. (3545)

229 "Turkey (republic)"/ (0)

230 turkey.mp. not "turkey (bird)"/ (39242)

231 Turkmenistan/ (561)

232 Turkmenistan.mp. (668)

233 or/207-232 (89175)

234 Argentina/ (12544)

235 Argentina.mp. (16179)

236 Belize/ (494)

237 (Belize or British Honduras).mp. (696)

238 Brazil/ (67481)

239 Brazil.mp. (80675)

240 Colombia/ (7334)

241 Colombia.mp. (9391)

242 Costa Rica/ (3118)

243 Costa Rica.mp. (4158)

244 Cuba/ (4575)

245 Cuba.mp. (5652)

246 Dominica/ (82)

247 Dominica.mp. (337)

248 Dominican Republic/ (1277)

249 Dominican Republic.mp. (1768)

250 Ecuador/ (2735)

251 Ecuador.mp. (3559)

252 Grenada/ (108)

253 Grenada.mp. (251)

254 Jamaica/ (3260)

255 Jamaica.mp. (3971)

256 Mexico/ (31414)

257 Mexico.mp. (44473)

258 Panama/ (1994)

259 Panama.mp. (3462)

260 Peru/ (6548)

261 Peru.mp. (8860)

262 Saint Lucia/ (62)

263 (St Lucia or Saint Lucia).mp. (277)

264 "Saint Vincent and the Grenadines"/ (47)

265 Grenadines.mp. (73)

266 Suriname/ (799)

267 Suriname.mp. (913)

268 Venezuela/ (4480)

269 Venezuela.mp. (5858)

270 or/234-269 (179573)

271 Algeria/ (2432)

272 Algeria.mp. (2979)

273 Iran/ (18129)

274 Iran.mp. (21107)

275 Iraq/ (4040)

276 Iraq.mp. (7389)

277 Jordan/ (3130)

278 Jordan.mp. (4481)

279 Lebanon/ (3328)

280 Lebanon.mp. (4068)

281 Libyan Arab Jamahiriya/ (0)

282 (Libya or libyan arab jamahiriya).mp. (1233)

283 Tunisia/ (6621)

284 Tunisia.mp. (7689)

285 or/271-284 (47421)

286 maldives/ (677)

287 Maldives.mp. (182)

288 or/286-287 (776)

289 Angola/ (764)

290 Angola.mp. (1096)

291 Botswana/ (1343)

292 (Botswana or Bechuanaland or Kalahari).mp. (1839)

293 Gabon/ (1279)

294 (Gabon or Gabonese Republic).mp. (1681)

295 Mauritius/ (504)

296 (Mauritius or Agalega Islands).mp. (806)

297 Namibia/ (824)

298 Namibia.mp. (1167)

299 Seychelles/ (319)

300 Seychelles.mp. (552)

301 South Africa/ (34686)

302 South Africa.mp. (39618)

303 or/289-302 (45915)

304 8 or 15 or 18 or 21 or 81 or 110 or 126 or 141 or 153 or 162 or 189 or 206 or 233 or 270 or 285 or 288 or 303

(949200)

305 exp vaccination/ (72946)

306 exp mass immunization/ or exp immunization/ (156727)

307 (immuni?e or immuni?ation).mp. [mp=title, abstract, original title, name of substance word, subject heading

word, keyword heading word, protocol supplementary concept word, rare disease supplementary concept word, unique

identifier] (140050)

308 (vaccine or vaccinate or vaccination).mp. [mp=title, abstract, original title, name of substance word, subject

heading word, keyword heading word, protocol supplementary concept word, rare disease supplementary concept word, unique

identifier] (231027)

309 305 or 306 or 307 or 308 (322712)

310 exp urban area/ or exp urban population/ (52689)

311 (urban or urbani?ation or slum or city or settlement).mp. [mp=title, abstract, original title, name of substance

word, subject heading word, keyword heading word, protocol supplementary concept word, rare disease supplementary

concept word, unique identifier] (222761)

312 310 or 311 (222761)

313 304 and 309 and 312 (2619)

314 limit 313 to yr="2000 -Current" (1659)
